# Exceptional Enlargement of the Mitochondrial Genome Results from Distinct Causes in Different Rain Frogs (Anura: Brevicipitidae: *Breviceps*)

**DOI:** 10.1155/2020/6540343

**Published:** 2020-01-22

**Authors:** Keitaro Hemmi, Ryosuke Kakehashi, Chiaki Kambayashi, Louis Du Preez, Leslie Minter, Nobuaki Furuno, Atsushi Kurabayashi

**Affiliations:** ^1^Amphibian Research Center, Hiroshima University, Hiroshima 739-8526, Japan; ^2^Faculty of Bio-Science, Nagahama Institute of Bio-Science and Technology, Shiga 526-0829, Japan; ^3^Unit for Environmental Sciences and Management, North-West University, Potchefstroom 2520, South Africa; ^4^South African Institute for Aquatic Biodiversity, Somerset Street, Grahamstown 6139, South Africa

## Abstract

The mitochondrial (mt) genome of the bushveld rain frog (*Breviceps adspersus*, Brevicipitidae, Afrobatrachia) is the largest (28.8 kbp) among the vertebrates investigated to date. The major cause of genome size enlargement in this species is the duplication of multiple genomic regions. To investigate the evolutionary lineage, timing, and process of mt genome enlargement, we sequenced the complete mtDNAs of two congeneric rain frogs, *B. mossambicus* and *B. poweri*. The mt genomic organization, gene content, and gene arrangements of these two rain frogs are very similar to each other but differ from those of *B. adspersus*. The *B. mossambicus* mt genome (22.5 kbp) does not differ significantly from that of most other afrobatrachians. In contrast, the *B. poweri* mtDNA (28.1 kbp) is considerably larger: currently the second largest among vertebrates, after *B. adspersus.* The main causes of genome enlargement differ among *Breviceps* species. Unusual elongation (12.5 kbp) of the control region (CR), a single major noncoding region of the vertebrate mt genome, is responsible for the extremely large mt genome in *B. poweri*. Based on the current *Breviceps* phylogeny and estimated divergence age, it can be concluded that the genome enlargements occurred independently in each species lineage within relatively short periods. Furthermore, a high nucleotide substitution rate and relaxation of selective pressures, which are considered to be involved in changes in genome size, were also detected in afrobatrachian lineages. Our results suggest that these factors were not direct causes but may have indirectly affected mt genome enlargements in *Breviceps*.

## 1. Introduction

Mitochondrial (mt) genomes of multicellular animals (metazoans) are generally closed-circular and double-stranded DNA molecules approximately 14–20 kbp in length [1–3]. However, genome size ranges from 6 to 48 kbp [2, 4], and linear and fragmented mtDNAs have been found in approximately 9000 animals investigated thus far [5–7]. In most metazoans, this small organelle genome encodes a typical set of 37 genes: 13 protein genes, involved in the electron transport system of respiration, two rRNA genes (*rrn*s), and 22 tRNA genes (*trn*s). In addition, animal mt genomes contain one long noncoding region, harboring several sequence elements related to mt genomic transcription and replication, named the control region (CR) or alternatively the D-loop region [2].

The mt genomic structure of metazoans, particularly vertebrates, tends to be conserved among closely related taxa. The same mt gene content and gene arrangement (synteny) are observed from fishes to mammals (e.g., [8, 9]). This genomic organization would have arisen in a common ancestor of vertebrates and has been maintained in a wide range of vertebrate taxa over 400 million years [10, 11].

However, in some vertebrate taxa, increases in gene content due to gene duplication and rearrangements of mt gene positions are often observed (e.g., [8, 12]). In particular, a greater degree of structural change in mt genomes has been reported for amphibians, especially modern anurans (neobatrachians) (e.g., [13, 14]). Among anurans, the members of the basal group (a paraphyletic group generally called the “Archaeobatrachia”) retain the typical ancestral (i.e., symplesiomorphic) mt genome organization of vertebrates [15, 16]. In contrast, most of the neobatrachians share the derived positions of three *trn*s translocated from their original locations (LTPF-*trn* cluster) [15, 17]. The Neobatrachia contains two superfamilies, Hyloidea and Ranoidea. The latter includes three large groups, Microhylidae, Natatanura, and Afrobatrachia [18, 19]. Of these, microhylid frogs retain the mt genomic structure of typical neobatrachian, while in natatanuran and afrobatrachian taxa, mt genomic rearrangements accompanying duplications and translocations of mt genes and the CR are often observed [13, 14, 16, 20, 21]. In particular, the mt genomes of afrobatrachians show large-scale structural changes.

The Afrobatrachia is a group of frogs, endemic to Africa, which currently consists of 422 described species in four families: Arthroleptidae, Brevicipitidae, Hemisotidae, and Hyperoliidae [22]. We have sequenced the complete mt genomes of four afrobatrachians representing all four afrobatrachian families [23]. All of these frogs tend to have large mt genomes, exceeding 20 kbp, and three of them have duplicated mt genes and/or CRs (excluding *Hemisus*). In particular, the mt genome of *Breviceps adspersus* (Brevicipitidae) has a highly reconstructed mt genome with many gene rearrangements and many duplicated gene regions. Consequently, the size of the *B. adspersus* mt genome is the largest among those vertebrates for which mtDNA has been sequenced to date (also the 13th largest among metazoans) [23].

To investigate the evolutionary origin and significance of the unusually large mt genome of *B. adspersus*, we analyzed the mt genomes of two additional *Breviceps* species, *B. mossambicus* and *B. poweri*. It has been suggested that the duplication of mt genes and the CRs, which results in an increase in genome size, are the result of nonadaptive evolution which, in insects, is correlated with an acceleration of nucleotide substitution rate [24] and a relaxation of purifying selective pressure, leading to a reduction in functional constraints that purge slightly deleterious mutations [25]. Thus, we investigated the changes in the substitution rate and selection pressure among afrobatrachian mt genomes and discuss the correlation between mt genome size and the change in the substitution rate and selective pressure.

## 2. Materials and Methods

### 2.1. Specimens Used

In this study, we used four frog specimens: one Mozambique rain frog, *Breviceps mossambicus* (Peters, 1854), and three Power's rain frogs, *B. poweri* (Parker, 1934) [26, 27]. These frogs were obtained via the pet trade; thus, their collection sites are unknown. The frog specimens were stored in 95.5% ethanol as part of AK's personal amphibian collection. The voucher numbers are 15-004, 15-008, and 15-010 for *B. poweri* and 14-001 for *B. mossambicus*.

### 2.2. Molecular Experiments

We extracted and purified the total DNA from the liver tissue of each fixed specimen with a DNeasy Blood & Tissue Kit (QIAGEN K. K., Tokyo, Japan) or using phenol/chloroform extraction with “DNA sui-sui” extraction buffer (Rizo Inc., Tsukuba, Japan) and ethanol precipitation methods [28].

From the purified total DNA, the whole mtDNA was amplified by PCR and sequenced for *B. poweri* (voucher 15-004) and *B. mossambicus*. The PCR amplification and sequencing procedures from Kurabayashi et al. [10] were followed. Specifically, for each specimen, we amplified 10 overlapping fragments containing the entire mt genome using the long and accurate (LA) PCR method with LA-Taq (Takara Bio Inc., Shiga, Japan) according to the manufacturer's instructions. These fragments were purified using the gel extraction method with a GenElute Agarose Spin Column (Sigma-Aldrich Japan Inc., Tokyo, Japan). The purified PCR fragments were sequenced using the primer walking method with an ABI 3130xl automated DNA sequencer (Applied Biosystems, Foster City, CA, USA). In this study, we used a total of 117 PCR primers for mtDNA amplification and/or sequencing, and 108 of them were newly designed during this study. All the primers used are listed in Supplementary [Supplementary-material supplementary-material-1].

### 2.3. NGS

The PCR fragments containing the CRs were very long, and these fragments harbored many direct repeat sequences that could not be read by the primer walking method (see Supplementary Fig. [Supplementary-material supplementary-material-1]). Thus, these fragments were sequenced using single-molecule real-time (SMRT) sequencing with the PacBio RS II next-generation sequencer (NGS), which allows exceptionally long read sequencing (max length per read > 40 kbp) [29]. We also applied the multiplex-amplicon approach (see [29]). Briefly, we amplified the CR fragments from three *B. poweri* and one *B. mossambicus* specimens (approx. 13 and 8 kbp, respectively; see Supplementary Fig. [Supplementary-material supplementary-material-1]) with the primers having distinct 3′ tag sequences for each specimen (consequently, the PCR fragments amplified from the same specimen have the same specific tag sequences, and thus, the PCR results were sortable from the mass NGS output-multiplex-amplicon method). Approximately 5 *μ*g of the gel-purified CR fragments of two *B. poweri* (vouchers 15-004 and 15-010) and one *B. mossambicus* was used for the library construction for NGS. We outsourced the library construction and SMRT sequencing to CoMIT (Center of Medical Innovation and Translational Research) of Osaka University. The first SMRT run allowed for the determination of the entire CR sequences of *B. mossambicus* and one *B. poweri* specimen (15-010). However, we could not obtain enough sequence reads for another *B. poweri* specimen (15-004). Thus, for this specimen, we made two internal PCR primers (named bfCSB_Fow1 and bfCSB_Rev2, Supplementary [Supplementary-material supplementary-material-1]) based on the resultant CR sequence of *B. poweri* (15-010). Using these primers, two fragments of the 5′ and 3′ sides of the CR (approx. 4 and 9 kbp, respectively) were separately amplified from *B. poweri* (15-004) and the fragments were sequenced by another SMRT run. The assembled sequences of each CR fragment were reconstructed from the RS II outputs using the Long Amplicon Analysis program implemented in the SMRT Link analysis system [29].

The assembled whole mtDNA sequences of *B. mossambicus* and *B. poweri* (15-004) and the CR sequence of *B. poweri* (15-010) were deposited in the International Nucleotide Sequence Database Collaboration (INSDC) under the accession numbers LC498571, LC498572, and LC498573, respectively.

### 2.4. Phylogenetic Analyses and Divergence Time Estimation

We performed phylogenetic tree reconstructions and a divergence time estimation by adding the sequence data obtained in this study to the dataset of Kurabayashi and Sumida [23], and the analytical methods used by Irisarri et al. [15] were followed. The previous dataset includes not only mt gene sequences but also nuclear genes. The nuclear data were also used in this study. Mitochondrial sequence data of afrobatrachians reported by Zhang et al. [16] were not used here because of a lack of genome size information and sequences of some mt genes. The genes used and their accession numbers are listed in Supplementary [Supplementary-material supplementary-material-1].

First, we aligned the sequences of each of the 13 protein genes, two *rrn*s, and 22 *trn*s, separately. The protein and RNA genes were aligned using the TranslatorX program with the default setting [30] and MAFFT with the L-INS-i option [31], respectively. The sequences of *trn*s were manually aligned using their secondary structures as a guide. Ambiguously aligned sites were deleted using the Gblocks program ver. 0.91b with the default parameters [32]. The final alignment dataset consisted of 21,063 bp (consisting of 13,938 and 7125 mt and nuclear gene sequences, respectively) from 49 operational taxonomic units (OTUs). The alignment data used are provided in Supplementary [Supplementary-material supplementary-material-1].

We used the following sequences in the phylogenetic analyses: two mt *rrn*s, 22 mt *trn*s, and the 1st and 2nd codons of the 13 mt and nine nuclear protein genes (total 15,093 bp). We did not use the 3rd codon positions of the protein genes in the phylogenetic analyses (and divergence time estimation) because it is known that their fast substitution rates could distort the reconstruction of deep anuran phylogenies [33, 34]. We also applied a partitioning strategy in the phylogenetic analyses; i.e., the concatenated sequence data were partitioned into statistically suitable partitions and a distinct nucleotide substitution model was applied for each sequence partition. The PartitionFinder program ver. 1.1.1 [35] was used to estimate the best partitioning scheme using Akaike's information criterion (AIC) [36]. Consequently, a setting with 17 distinct partitions was suggested as the best partitioning scheme and this partitioning scheme was used in both the maximum likelihood (ML) and Bayesian inference (BI) analyses. An independent general time reversible+gamma distribution (GTR+G) substitution model for each of the 17 partitions was applied in the ML analysis. For the BI analysis, the best substitution model was estimated for each partition using the Kakusan4 program [37]. The detailed partitioning scheme and the suggested substitution models in BI are summarized in Supplementary [Supplementary-material supplementary-material-1].

We performed phylogenetic reconstructions using ML and BI methods. RAxML ver. 8.2.12 and MrBayes ver. 3.2.6 software packages were used for the ML and BI analyses, respectively [38, 39]. The rapid hill climbing algorithm (implemented in RAxML) with the starting tree option of 100 randomized parsimonious trees was applied for the ML analysis. For the BI analysis, 10 million generations of four Markov chains (MCs) were run with one sampling per every 1000 generations and the 1st 10% samples were discarded as burn-in. The convergence of the posterior distribution of model parameters (all parameters reached >200) was checked using Tracer ver. 1.5 [40]. The supports for the internal branches of reconstructed trees were evaluated using bootstrap percentages (BPs) calculated by 1000 pseudoreplications and Bayesian postprobabilities (BPPs) in ML and BI analyses, respectively.

**Figure 1:**
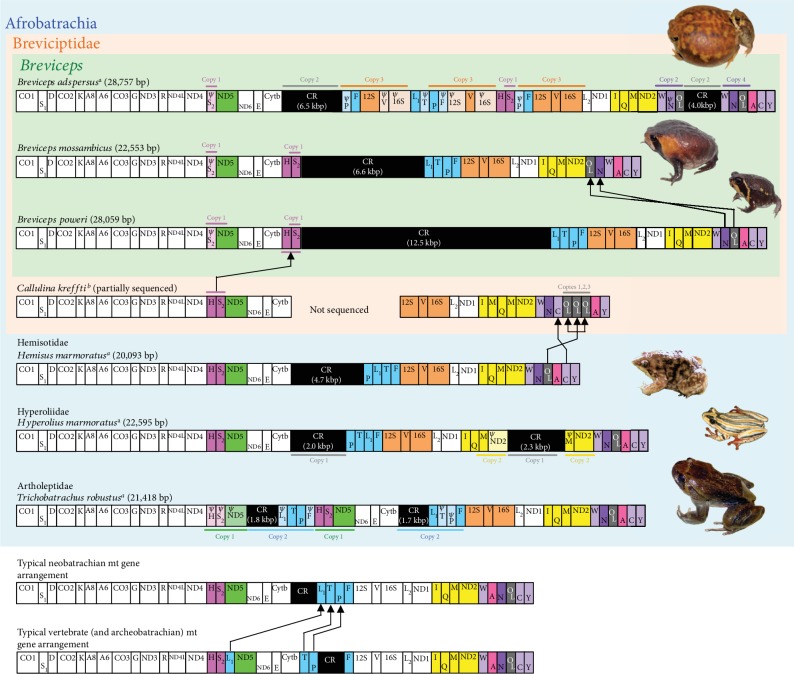
Mitochondrial genome organization of afrobatrachians and other anurans. The mitochondrial (mt) genome organization of *Breviceps mossambicus* and *B. poweri* determined in this study is compared with that of other afrobatrachians, neobatrachians, and vertebrates reported in previous studies (^a^Kurabayashi and Sumida [23] and ^b^Zhang et al. [16]). Genes, pseudogenes, control regions (CRs), and *light*-strand replication origins (O_L_) are shown in boxes. The *heavy*- and *light*-strand encoded genes are denoted above and below each gene box, respectively. The boxes do not reflect the actual sizes of the genes and CRs. The single-letter amino acid codes designate the corresponding transfer RNA genes (*trn*s). L1, L2, S1, and S2 indicate *trn*s for Leu (UUR), Leu (CUN), Ser (UCN), and Ser (AGY), respectively. “*ψ*” shows a pseudogene. Other gene abbreviations are as follows. 12S and 16S: 12S and 16S ribosomal RNAs; CO1–3: cytochrome c oxidase subunits 1–3; Cytb: cytochrome apoenzyme *b*; ND1–6 and 4L: NADH dehydrogenase subunits 1–6 and 4L. The genes, pseudogenes, O_L_, and CRs with duplications and/or rearrangements in afrobatrachians are colored. “Copy” with number shows the duplicated regions within a species. Closed arrows between species indicate the rearranged genes and the presumed evolutionary direction of the translocations. The photos of afrobatrachian species are also provided (excluding *Callulina kreffti*).

The divergence times of anurans were estimated using a Bayesian dating method with the BEAST ver. 2.5.2 program package [41]. In the estimation, the amphibian phylogenies recovered from both ML and BI analyses were used as the topology constraint (Figure 1). The sauropsid monophyly (lizard+bird), not recovered by our ML and BI analyses, was *a priori* assumed in this dating analysis as in previous studies [15, 23]. The same data partitioning scheme and substitution models used in the BI analysis were also applied here. We used the Yule process [42] to describe cladogenesis. The final MCs were run twice for 100 million generations with one sampling per every 10,000 generations, and the 1st one million generations were discarded as burn-in. The posterior distributions of model parameters were checked in the same way as the above BI analysis. Following Irisarri et al. [15], we applied seven (lower age boundaries) calibration points as follows: (A) Sauropsida-Synapsida split: >312 million years ago (Ma), (B) Archosauromorpha-Lepidosauromorpha split: >260 Ma, (C) Cryptobranchidae-Hynobiidae split: >146 Ma, (D) Anura-Caudata split: >249 Ma, (E) most recent common ancestor (MCA) of Discoglossoidea: >161 Ma, (F) MCA of Pipoidea: >146 Ma, and (G) Calyptocephalella-Lechriodus split: >53 Ma. These were used as prior boundaries for divergence time estimation.

### 2.5. Relative Rate Test

We compared the relative rates of nucleotide substitutions of mt genes (all mt genes, all mt protein-coding genes, all *rrn*s, and all *trn*s) among afrobatrachian lineages using relative rate tests (RRTs) [43] with the RRTree program [44]. The Kimura two-parameter substitution model [45] was used for the estimation of genetic distances. In this analysis, we used the gene data of 24 neobatrachians. Noncompared lineages were used as the outgroups in each comparison (e.g., when we compared *Hemisus* and the three *Breviceps* species, the lineages of the remaining 20 neobatrachians were regarded as the outgroups). The lineages for each comparison are shown in Table 1.

### 2.6. Detection of Changes in Lineage-Specific Selective Pressure

It is known that the ratio of nonsynonymous/synonymous substitutions (dN/dS ratio = *ω*) can be used to identify the changes in selective pressure among taxa [15, 46, 47]. To understand the changes in the dN/dS ratio in ranoid lineages, we used the “branch model” analysis [48] using the Codeml program implemented in PAML 4.9 [49]. In this analysis, we used the alignment data of mt protein genes from 24 neobatrachian taxa and the ML and BI tree topology. We compared five branch models with distinct assumptions about dN/dS ratios. In one model (null model), the single constant *ω* was assumed in all neobatrachian lineages while other models allowed the changes in the dN/dS ratios on specific ranoid branches. The details of the models are shown in Table 2. The branch lengths were first calculated under the null model, and the resultant branch lengths were applied in the other models. The significance of the differences in log likelihoods among these models was tested using the likelihood ratio test (LRT).

## 3. Results and Discussion

### 3.1. Huge mt Genomes of Rain Frogs

We determined the entire mt DNA sequences of two rain frogs, *B. mossambicus* and *B. poweri*. These mt genomes are 22,553 and 28,059 bp in length, which are very large for a vertebrate mt genome (typical size range is 16–17 kbp [1]). Although the genome size of *B. mossambicus* is similar to those of other afrobatrachian frogs (e.g., marbled reed frog (*Hyperolius marmoratus*): 22,595 bp and hairy frog (*Trichobatrachus robustus*): 21,418 bp; see Figure 1), the *B. poweri* mt genome was the second largest among the vertebrates investigated thus far (according to NCBI organelle genome resources [7] as of August 2019). The third largest vertebrate mt genome is 25,972 bp for the prickly gecko (*Heteronotia binoei*) [50]. Therefore, the mt genome of *B. poweri* is closer in size to that of *B. adspersus*, which possesses the largest known mt genome of vertebrates (28,757 bp).

In contrast to the large genome sizes, the mt gene content of both *B. mossambicus* and *B. poweri* is similar to that of many other vertebrates (Figure 1), containing the set of 37 single genes (13 proteins, two rRNA, and 22 tRNA genes) and single long and short noncoding regions commonly found in vertebrate mt genomes. The long noncoding region is referred to as CR and contains putative sequence elements involved in DNA replication and RNA transcription (e.g., terminate associate sequence (TAS) and conserved sequence blocks 1–3 (CSB1–3); see Supplementary Fig. [Supplementary-material supplementary-material-1] and [51]), and a short noncoding region is known as the *light*-strand replication origin (O_L_). The presence of a small pseudogene, *trn*S(AGY), between NADH dehydrogenase subunits 4 and 5 (*nd4* and *nd5*) is the singular exception in gene content (Figure 1).

The gene content of *B. mossambicus* and *B. poweri* is not similar to that of the congeneric *B. adspersus* mt genome, which has many duplicated and triplicated gene regions and duplicated CRs, making it the largest known mt genome among vertebrates (Figure 1). Instead, the cause of the mt genome enlargement in *B. mossambicus* and *B. poweri* is the unusual expansions of the control regions. The lengths of their CRs are 6,618 and 12,537 bps, respectively, although the typical CR lengths in vertebrates are 1-2 kbp [1]. The long CRs of *B. poweri* and *B. mossambicus* result from the occurrence of multiple and long-tandem repeats (Supplementary Fig. [Supplementary-material supplementary-material-1]). The *B. mossambicus* CR contains four distinct direct tandem repeats, and these repetitive sequences, totaling 4228 bp in length, occupy 63.9% of the CR. The CR of *B. poweri* (individual 4) has six distinct repeat sequence groups. Two of them are quite long (see Supplementary Fig. [Supplementary-material supplementary-material-1]): one repeat group consists of 1,150 and 1,151 bp, nearly complete, repeat sequences (repeat 1: totaling 2301 bp), while another is composed of 23 units of 233–405 bp incomplete direct repeat sequences (repeat 3: totaling 7,339 bp). Consequently, the total length of the six repeat sequences in the *B. poweri* CR is 10,625 bp (occupying 84.7% of the CR nucleotides). The long CR over 10 kbp in length is quite rare and has never been completely sequenced in other vertebrate taxa (approximately 12 kbp CR occurred in a Malagasy frog (*Gephyromantis pseudoasper*) [20]).

To determine whether the unusually long CR is specific to the individual frogs used or is a common characteristic of the frog species, we checked the CRs of two additional *B. poweri* individuals. The PCR fragments having similar lengths (approx. 13 kbp, including the whole CR and 5′and 3′ franking gene regions) are commonly amplified in all three individuals (Supplementary Fig. [Supplementary-material supplementary-material-1]). We also sequenced the CR of one of the two additional individuals. Although the CR lengths differ by 627 bp between the *B. poweri* individuals (12,537 bp vs. 11,910 bp), the CR sequences of the two *B. poweri* individuals are quite similar, excluding the number of repeat units. Therefore, the very long CR, with numerous repetitive sequences, seems to be a common feature of this rain frog species.

It is almost impossible to precisely sequence long repetitive regions exceeding 10 kbp, such as those of the *B. poweri* CR by conventional sequencing methods (i.e., primer walking and construction of deletion mutants [20]) or by using the NGS technique with a short-read sequencing strategy [52]. In this study, a long-read strategy with PacBio RS II allowed us to relatively easily sequence such long repetitive sequences. These results demonstrate the superiority and necessity of long-read sequencing in analyzing the repetitive sequences occasionally found in both organelles and nuclear genomes.

### 3.2. Phylogeny, Timing, and Distinct Causes of mt Genome Enlargement in These Congeneric Rain Frogs

**Figure 2:**
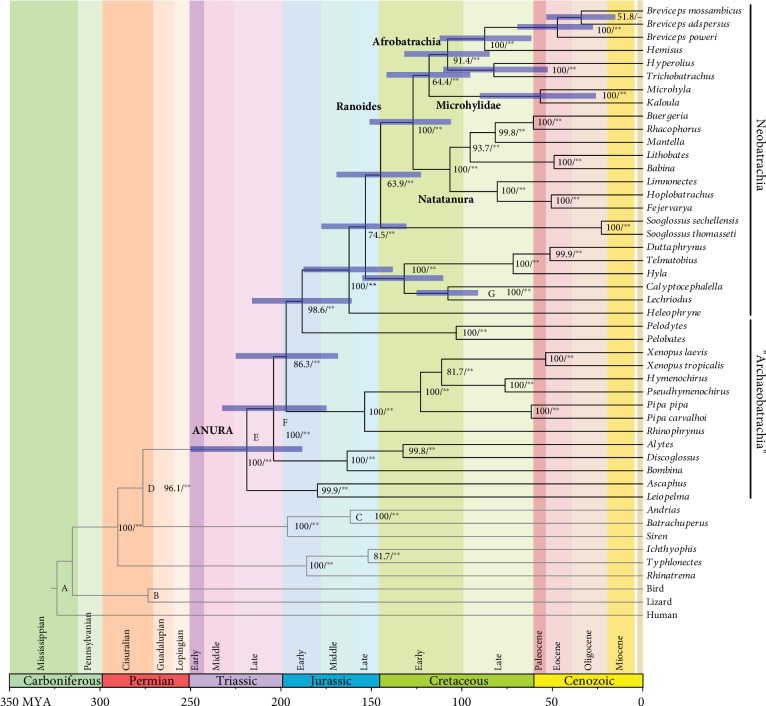
Time tree of anurans. A phylogenetic tree reflecting the divergence ages estimated using a Bayesian relaxed dating method with the 15,093 bp nucleotide data. The tree topology of amphibians is the same as that of the resultant ML and BI trees. Bold branches indicate the lineages leading to the extant anurans. Horizontal blue bars on each node indicate 95% credibility intervals of estimated divergence age. The bootstrap probability (BP) of ML and Bayesian postprobabilities (BPP) are also shown on the right side of each node (BP value/^∗^, ^∗∗^ > 95 and 99 BPPs), and the calibration points used in the dating analysis are indicated on the corresponding nodes (A to G). The scale of the horizontal axis is in million years.

To infer the phylogenetic lineages and evolutionary times of the mt genome enlargement of afrobatrachians, including *Breviceps*, we performed molecular phylogenetic analyses and divergence time estimation. The ML and BI methods reconstructed the same tree topology, and the maximum log likelihood (ln*L*) of the resultant ML and the average ln*L* of the BI tree were -224667.83 and -221797.17, respectively. The time tree of amphibians having the ML/BI tree topology is shown in Figure 2. The major anuran phylogenies and the split ages agreed completely or to a substantial degree with those from previous studies: e.g., the monophyly of neobatrachians and paraphyly of archaeobatrachians with respect to neobatrachians (most recent common ancestor of Neobatrachia = 162.3 Ma), monophyly of ranoids (MCA = 127.0 Ma), and the three major clades of ranoids (Natatanura, Microhylidae, and Afrobatrachia, which started to split from 127.0 Ma) (e.g., [15, 16, 19, 53]).

**Figure 3:**
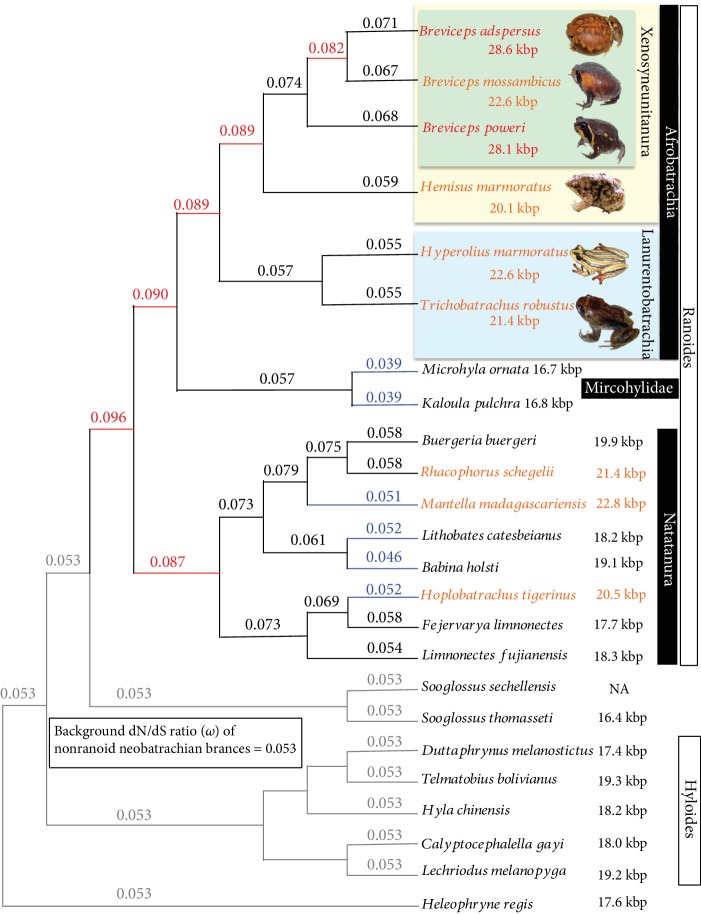
Changes in the dN/dS ratio (*ω*) among ranoid lineages. The estimated *ω* values of neobatrachian branches are shown (based on model 4 in Table 2). The tree topology is the same as those of the ML and BI trees reconstructed in this study. The constant *ω* (0.053) of nonranoid neobatrachian lineages was regarded as the background value. The estimated *ω* is shown on each ranoid branch. A high *ω* indicates the relaxation of purifying pressure. The branches for which *ω* values are lower (<0.053) and 1.5 times higher (>0.08) than the background are shown in blue and red colors, respectively. The frog taxa having mtDNAs exceeding >20 kbp and 28 kbp are also highlighted by orange and red colors, respectively.

The resultant phylogenies of afrobatrachian taxa completely match those of recent studies [16, 23, 54] (Figure 3), excluding the Afrobatrachia+Microhylidae clade not supported in Feng et al. [19]. Specifically, (1) Afrobatrachia is monophyletic, (2) Microhylidae becomes the sister taxon of Afrobatrachia, (3) Brevicipitidae forms a clade with Hemisotidae (this clade is called Xenosyneunitanura [18]), (4) *Breviceps* is monophyletic and *B. poweri* is the basal taxon of the three species sampled in this genus, and (5) *B. mossambicus* and *B. adspersus* have a closer affinity within this sample. The estimated divergence ages of the corresponding nodes are as follows: (1) 118.1 Ma for the divergence between Afrobatrachia and Microhylidae, (2) 107.9 Ma for the most recent common ancestor of afrobatrachians, (3) 87.4 Ma for the split of Brevicipitidae and Hemisotidae, (4) 47.1 Ma for the divergence of *B. poweri* from the lineage of *B. mossambicus*+*B. adspersus*, and (5) 34.0 Ma for the split of *B. mossambicus* and *B. adspersus*. The divergence ages estimated here largely agree with those from recent studies [16, 23]. However, for the *Breviceps* divergences, younger ages have been estimated by Nielsen et al. [54] (20 and 18 Ma for the divergences of 4 and 5, respectively). They used a total of 24 *Breviceps* taxa and applied some young calibration points within afrobatrachian lineages in their dating analyses. These differences in the data analysis could account for the differences in estimated divergence ages between our study and that of Nielsen et al. [54].

As described above, the mt genomes of microhylid frogs, the sister group of Afrobatrachia, exhibit average genome sizes for vertebrates (16.7–17.2 kbp) [55, 56]. In contrast, the afrobatrachian mt genomes exceed 20 kbp for all six species examined. Thus, the mt genome enlargement appears to be an evolutionary trend that has arisen in the lineages leading to the living afrobatrachians after the split from the microhylid lineage (Figures 2 and 3). In particular, the mt genome sizes of the two *Breviceps* species, *B. adspersus* and *B. poweri*, are over 28 kbp, making them as the largest and second largest known mt genomes of vertebrates. With regard to the interspecific phylogeny of *Breviceps*, these two species are not monophyletic; i.e., *B. adspersus* is closer to *B. mossambicus* than to *B. poweri* (Figure 1). Overall, our results indicate that the huge mt genomes arose in two independent rain frog lineages within relatively short periods (>47 and >30 million years for the lineages leading to *B. poweri* and *B. adspersus*, respectively). Furthermore, it is noteworthy that the physical causes of mt genome enlargement differ between these two rain frogs. The duplication of multiple gene/CRs and the accumulation of repetitive sequences are the main causes of mt genome enlargement in *B. adspersus* and *B. poweri*, respectively.

There are several examples of mt genome enlargement shared by congeneric species. For example, Malagasy poison frogs (*Mantella* spp.) commonly have >22 kbp mt genomes enlarged by the duplication of genes and CRs [20, 25], and two *Scapharca* ark shells exhibit >46 kbp mt genomes, mainly caused by elongations (>30 kbp) of noncoding sequences [4, 57]. However, there has been no similar example to that of *Breviceps* where the mt genome enlargement occurs in independent congeneric lineages, from distinct causes. Thus, the mt genomic structure is highly variable in this frog taxon.

### 3.3. Gene Rearrangements and Evolutionary Inference

Although a highly rearranged mt genome is present in *B. adspersus* [23], the mt gene content and their arrangements of the congeneric *B. poweri* and *B. mossambicus* do not deviate largely from the typical mt genomes of vertebrates (Figure 1). The latter *Breviceps* species have almost the same gene arrangement, with the exception of two small translocations of *trn*N and light-strand replication origin (O_L_) within the WN-O_L_-ACY *trn* cluster (Figure 1). Because the order of *trn*sWN-O_L_ found in *B. poweri* (and also in *B. adspersus*) is a primitive (plesiomorphic) gene order commonly shared by other afrobatrachians (Figure 1) [16, 23], the O_L_-*trn*sNW order of *B. mossambicus* appears to be newly emerged in the lineage leading to this species.

The *B. poweri* and *B. mossambicus* mt gene arrangements are similar to those of the mt gene orders of neobatrachians, especially *Hemisus*. Excluding the two minor *trn* translocations that occurred within the same *trn* clusters (specifically O_L_-*trn*sNW in *B. mossambicus* and *trn*sPLTF in *Hemisus*), the translocation of *trn*sHS is the only distinctive difference between *Breviceps* and *Hemisus*. Although the *trn*sHS is located between *nd4* and *nd5* in most neobatrachians, including *Hemisus*, this *trn* block lies between the cytochrome apoenzyme *b* gene (*cytb*) and CR in *B. poweri* and *B. mossambicus*. The novel *cytb*-*trn*sHS-CR arrangement could have arisen in a common ancestor of *Breviceps* after the split with other brevicipitid genera, for the following reasons: (1) the original *nd4*-*trn*sHS-*nd5* arrangement remains in another brevicipitid, *Callulina kreffti*, mt genome [16] and (2) the pseudogene of *trn*S is found at its original position between *nd4* and *nd5* in *B. poweri* and *B. mossambicus* (Figure 1).

The gene rearrangements in animal mt genomes are considered to reflect animal evolution (e.g., [23, 58–60]), and the rearranged gene orders found in this study can be regarded as novel phylogenetic markers for brevicipitid taxa. Specifically, the O_L_-*trn*sNW can be considered characteristic of some members of the *B. mossambicus* group [54], while the *cytb-trn*sHS-CR can be regarded as a synapomorphy of *Breviceps*.

In the *B. adspersus* mt genome, the *trn*sHS block is further translocated and positioned within the triplicated *trn*s-LTPF-12S*rrn*-*trn*V-16S*rrn* cluster. In addition, duplications and translocations of the *trn*sWN-OL cluster and CR were also found in this species (Figure 1) [23]. The detailed phylogenetic lineage and evolutionary period of these large genomic modifications have not been clarified. This study shows that the mt genomes of *B. poweri* and *B. mossambicus* are not markedly rearranged from the typical neobatrachian type, although *B. adspersus* branches between these species' lineages (Figure 1 and see [54]). Therefore, it can be concluded that the unique mt genome of *B. adspersus* evolved only in the lineage leading to this species. Large mt genomic modifications resulting in stepwise gene rearrangements along with multiple lineage splits have been reported in Malagasy frogs (Mantellidae) [20]. However, in *Breviceps*, large-scale genomic changes occurred in a single species lineage in a relatively short period (>47 Ma in this study and >20 Ma according to [54]), suggesting that the genomic structure of vertebrate mtDNA could harbor higher structural variability than is generally believed (e.g., [9]).

### 3.4. Substitution Rates and Selective Pressure on the Afrobatrachian mt Genomes and Their Correlations with Genome Size Expansion

As mentioned above, the duplication of genes/CRs and the occurrence of numerous repetitive sequences in the CR are responsible for the huge mt genomes of *B. adspersus* and *B. poweri*, respectively. Most of these events occurred in tandem, except for the nontandem duplications of CR and *trn*S(AGY) (Figure 1), which could have been caused by a nontandem duplication mechanism [20, 23]. In animal mt genomes, it is believed that both duplicated gene regions and tandem repeats in the CR have emerged because of errors in mtDNA replication, such as imprecise replication termination and strand slippage of the nascent DNA strand (e.g., [61, 62]). Furthermore, it has been suggested that accelerated nucleotide changes lead to the frequent tandem duplications via frequent substitutions at the initiation and termination points of mtDNA replication [24]. To investigate the correlation between genome size and nucleotide substitution rates, we compared the relative substitution rates of mt genes (four categories: all 37 genes, 13 protein genes, two *rrn*s, and 22 *trn*s) among afrobatrachian-related taxa using the RRT (Table 1).

The RRT showed that the substitution rates of afrobatrachian mt genes commonly having large genome sizes (>20 kbp) are significantly faster than those of the sister taxon, the microhylids, with normal genome sizes in all compared categories. Furthermore, the substitution rates of xenosyneunitanurans (Brevicipitidae+Hemisotidae), including the longest mt genomic species, are significantly higher than those of another afrobatrachian group, the laurentobatrachians (Arthroleptidae+Hyperoliidae) for all mt genes and protein genes (but are not significant for *rrn*s and *trn*s). These results suggest a correlation between the substitution rate and mt genome size. However, the substitution rates of the *Hemisus* mtDNA with a 20 kbp genome size are not significant but are faster than those of *Breviceps* species in all four compared categories. Among *Breviceps* species, the *B. poweri* mt genes show faster substitution rates compared to those of *B. mossambicus* and *B. adspersus*, although the latter species has the largest mt genome among vertebrates. From these results, it is concluded that mt genomes with large genome sizes also tend to have fast nucleotide substitution rates but the latter factor is not a direct cause of genome enlargement in afrobatrachian frogs.

It has been shown that animal mt genomes are subject to a strong purifying pressure that suppresses mutations leading to functional changes because the functions of mt coding genes are essential for respiration (e.g., [63]). Nevertheless, it has been reported that the purifying pressure of mt genomes is relaxed in neobatrachians, especially in the ranoid lineages [15, 23]. A relaxation of purifying selective pressure, leading to a reduction in functional constraints that purge slightly deleterious mutations, has been suggested as one of the causes of mt genome enlargement [25]. In general, the ratio of nonsynonymous and synonymous substitutions (dN/dS ratio, *ω*) is useful to understanding the conditions for selection of the genes: positive selection (*ω* > 1), neutral evolution (*ω* = 1), and purifying selection (*ω* < 1). Here, we estimate the dN/dS ratio using the branch model to investigate the changes in selective pressures in the ranoid lineages. In this analysis, we calculated the *ω* value(s) of the five branch models and compared the resultant log likelihood values among these models (Table 2). The LRT showed that model 4, with variable *ω* for all neobatrachian branches, is the best fitted among the models tested (*P* = 6.6 × 10^−21^ vs. model 3 with the 2nd largest likelihood value).

Figure 3 indicates the estimated *ω* values for the ranoid lineages under the best branch model (model 4). In all neobatrachian lineages, the estimated *ω* values are less than 1 (0.039–0.096) and confirm that the mt genomes of neobatrachian frogs are under strong purifying pressure. Compared to the background *ω* value (0.053) of the nonranoid neobatrachian lineage, the *ω* values are high in 25 of 31 ranoid branches (the branches shown in black and red in Figure 3), indicating that the purifying selection has been relaxed in these lineages. Largely relaxed branches mainly correspond to the ancestral lineages, specifically common ancestral lineages leading to *Breviceps* (i.e., ranoids, afrobatrachians, and microhylids, afrobatrachians, and xenosyneunitanurans: *ω* = 0.096, 0.090, 0.89, and 0.89, respectively). In contrast, the *ω* values of the branches leading to the extant frogs (i.e., terminal branches) tend to be lower. On the other hand, the *ω* values of six out of 16 terminal branches are less than the background *ω* value, indicating that the purifying selection increased again in these lineages.

Among anurans, the mt genomes with >20 kbp size have only been reported from ranoid taxa (see [7]) and obvious increases in the dN/dS ratio are found in most ranoid lineages. These facts appear to support the idea that the sizes of the mt genome can change concomitantly with lower selection strengths [25]. However, it is remarkable that reincreased purifying pressures are found in species lineages leading to *Mantella* and *Hoplobatrachus*, having >20 kbp mt genomes (*ω* = 0.051 and 0.052, respectively). Furthermore, selective pressures of the species lineages of *B. adspersus* and *B. poweri* (*ω* = 0.071 and 0.068, respectively), i.e., the exact lineages with increased genome size, are not as relaxed as those of their ancestral lineages. Consequently, our findings suggest that low selective pressure does not directly cause the huge mt genomes as does substitution rate (see the above). Rather, the existence of relaxed purifying selection in the ancestral lineages seems to have an indirect effect on the mt genome size. This indirect correlation might be caused by the accelerated accumulation of deleterious mutations under low selective pressure. Significantly, the mutations occur at the DNA replication-related sites which could induce numerous repetitive regions, leading to large mt genomes, as pointed out in Shao et al. [24].

## 4. Conclusion

In this study, we show that *B. poweri* has the second largest known mt genome among the vertebrates after its congeneric *B. adspersus*. We also found that the unusually large mt genomes did not arise in a common ancestor of these *Breviceps* species but rather that the genome enlargement occurred independently in each species lineage within relatively short periods. Consistent with this evolutionary inference, the causes of the genome enlargement differed between these species. At present, 19 nominal species are known in *Breviceps* [22], but only three mt genomes have been analyzed. It is remarkable that two of these mt genomes are the largest known among the vertebrates. Also, the mt genomic structures differ significantly between these congeneric species, suggesting high variability in the mt genomic structures in this genus. Future investigations of *Breviceps* mt genomes may provide new insights into the hidden diversity of vertebrate mt genomes.

## Figures and Tables

**Table 1:** Comparisons of the relative rates of nucleotide substitutions among afrobatrachian-related lineages. The lineages with faster substitution rates are shown in bold.

Compared genes	Compared lineages		Relative substitution rates		Probability	Significance^∗∗^
	Lineage 1	Lineage 2	Lineage 1	Lineage 2		
Microhylids vs. Afrobatrachians						
All 37 mt genes	Microhylids	**Afrobatrachians**	0.340	**0.380**	1.0 × 10^−7^	^∗∗^
All 13 protein genes	Microhylids	**Afrobatrachians**	0.391	**0.428**	1.0 × 10^−7^	^∗∗^
All rRNA genes	Microhylids	**Afrobatrachians**	0.165	**0.204**	1.0 × 10^−7^	^∗∗^
All tRNA genes	Microhylids	**Afrobatrachians**	0.225	**0.286**	1.0 × 10^−7^	^∗∗^
Laurentobatrachia (Arthroleptidae+Hyperoliidae) vs. Xenosyneunitanura (Hemisotidae+Brevicipitidae)						
All 37 mt genes	Laurentobatrachians	**Xenosyneunitanurans**	0.343	**0.361**	2.1 × 10^−5^	^∗∗^
All 13 protein genes	Laurentobatrachians	**Xenosyneunitanurans**	0.389	**0.408**	2.6 × 10^−4^	^∗∗^
All rRNA genes	Laurentobatrachians	**Xenosyneunitanurans**	0.178	**0.192**	0.098	
All tRNA genes	Laurentobatrachians	**Xenosyneunitanurans**	0.248	**0.267**	0.114	
*Hemisus* vs. *Breviceps* (Hemisotidae vs. Brevicipitidae)						
All 37 mt genes	***Hemisus***	*Breviceps*	**0.369**	0.358	0.058	
All 13 protein genes	***Hemisus***	*Breviceps*	**0.413**	0.404	0.207	
All rRNA genes	***Hemisus***	*Breviceps*	**0.203**	0.184	0.083	
All tRNA genes	***Hemisus***	*Breviceps*	**0.279**	0.266	0.470	
*Breviceps mossambicus* vs. *B. adspersus*						
All 37 mt genes	*B. mossambicus*	***B. adspersus***	0.351	**0.351**	0.993	
All 13 protein genes	*B. mossambicus*	***B. adspersus***	0.393	**0.394**	0.906	
All rRNA genes	*B. mossambicus*	***B. adspersus***	0.185	**0.190**	0.514	
All tRNA genes	***B. mossambicus***	*B. adspersus*	**0.279**	0.266	0.237	
*Breviceps mossambicus* vs. *B. poweri*						
All 37 mt genes	*B. mossambicus*	***B. poweri***	0.351	**0.375**	4.6 × 10^−6^	^∗∗^
All 13 protein genes	*B. mossambicus*	***B. poweri***	0.393	**0.423**	5.9 × 10^−6^	^∗∗^
All rRNA genes	*B. mossambicus*	***B. poweri***	0.185	**0.193**	0.311	
All tRNA genes	*B. mossambicus*	***B. poweri***	0.279	**0.287**	0.551	
*Breviceps adspersus* vs. *B. poweri*						
All 37 mt genes	*B. adspersus*	***B. poweri***	0.351	**0.375**	3.7 × 10^−6^	^∗∗^
All 13 protein genes	*B. adspersus*	***B. poweri***	0.394	**0.423**	6.5 × 10^−6^	^∗∗^
All rRNA genes	*B. adspersus*	***B. poweri***	0.190	**0.193**	0.695	
All tRNA genes	*B. adspersus*	***B. poweri***	0.266	**0.287**	0.087	

^∗∗^<0.001.

**Table 2:** Branch models used to estimate the dN/dS ratio (*ω*) of ranoid lineages.

Model		Constraint of the model	−ln*L*^∗^ of the model	LRT^∗∗^ vs.			
				Model 0	1	2	3
0	Constant *ω*	All neobatrachian branches have single *ω*	148549.3	—	—	—	—
1	Two *ω* (Afrobatrachia)	Afrobatrachian branches have one unique *ω*	148536.2	3.2 × 10^−7^	—	—	—
2	Two *ω* (Ranoidea)	Ranoid branches have one unique *ω*	148539.0	5.5 × 10^−6^	NC^∗∗∗^	—	—
3	Eleven *ω*	All afrobatrachian branches have distinct *ω*	148515.9	5.1 × 10^−10^	1.3 × 10^−5^	1.4 × 10^−6^	—
4	Thirty-one *ω*	All ranoid branches have distinct *ω*	148443.6	9.6 × 10^−29^	2.7 × 10^−24^	2.6 × 10^−25^	6.6 × 10^−21^

^∗^ − ln*L*: minus log likelihood. ^∗∗^LRT: *P* value of the likelihood ratio test. ^∗∗∗^Models 1 and 2 have the same degrees of freedom (3) and cannot be compared by LRT. However, model 2 had a higher −ln*L*.

## Data Availability

The data used to support the findings of this study are available from the INSDC under the accession numbers LC498571, LC498572, and LC498573 and Supplementary [Supplementary-material supplementary-material-1].

## Supplementary Materials

Supplementary Materials Supplementary Table S1: list of primers used in this study. Supplementary Table S2: details on OTUs and gene data used. Supplementary Data S1: alignment data, partitioning strategy, and substitution models used in phylogenetic reconstructions and divergence time estimation. Supplementary Fig. S1: control regions of *Breviceps poweri* and *B. mossambicus*. Supplementary Fig. S2: PCR fragments of *Breviceps poweri* and *B. mossambicus* control regions.
