# Supplementary material for: Exceptional Enlargement of the Mitochondrial Genome Results from Distinct Causes in Different Rain Frogs (Anura: Brevicipitidae: Breviceps)
Source: Int J Genomics. 2020 Jan 22;2020:6540343. doi: 10.1155/2020/6540343 (PMC6998742; doi:10.1155/2020/6540343)
Supplement: Supplementary Materials — Supplementary Table S1: list of primers used in this study. Supplementary Table S2: details on OTUs and gene data used. Supplementary Data S1: alignment data, partitioning strategy, and substitution models used in phylogenetic reconstructions and divergence time estimation. Supplementary Fig. S1: control regions of Breviceps poweri and B. mossambicus. Supplementary Fig. S2: PCR fragments of Breviceps poweri and B. mossambicus control regions. [file 6540343.f1.zip › Suppl_Fig S2.pdf]

Supplementary Figure S2.
PCR fragments of *Breviceps poweri* and *B. mossambicus* control regions.

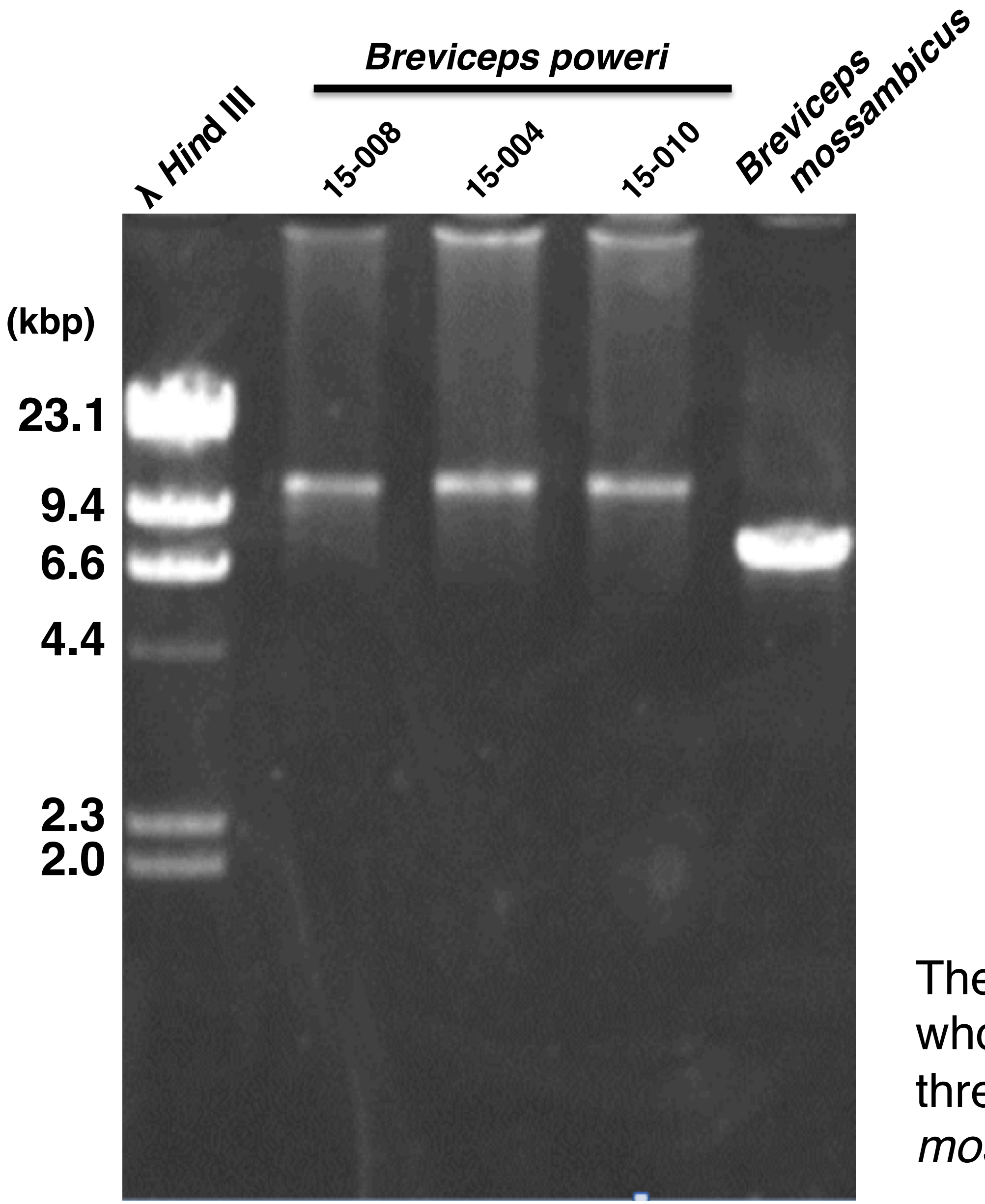

The PCR fragments containing the whole control regions amplified from three *Breviceps poweri* and one *B. mossanbicus* specimens are shown.
